# Machine-Learning-Assisted Discovery of Cr^3+^-Based Near-Infrared Phosphors

**DOI:** 10.1021/acs.chemmater.5c01208

**Published:** 2025-09-19

**Authors:** Amit Kumar, Arslan Akbar, Hannah Lesmes, Seán R. Kavanagh, David O. Scanlon, Jakoah Brgoch

**Affiliations:** 1 Department of Chemistry, 14743 University of Houston, Houston, Texas 77204, United States; 2 Texas Center for Superconductivity, 14743 University of Houston, Houston, Texas 77204, United States; 3 98029 Harvard University Center for the Environment, Cambridge, Massachusetts 02138, United States; 4 School of Chemistry, University of Birmingham, Birmingham B15 2TT, United Kingdom

## Abstract

Cr^3+^-substituted inorganic phosphors exhibit three distinct near-infrared (NIR) photoluminescence emission peak shapes that typically fall between 650 and 950 nm. The exact position and shape are governed by the (weak, intermediate, or strong) crystal field splitting environment of the octahedrally coordinated Cr^3+^ ions. These emission characteristics are commonly quantified by the Dq/*B* ratio, where Dq represents the crystal field splitting parameter and *B* is the Racah parameter. Precise knowledge of this ratio is therefore critical for designing Cr^3+^-based NIR phosphors for applications like biomedical imaging, night vision, food quality analysis, and luminescence thermometry. Unfortunately, targeting specific Dq/*B* values in the solid state remains nontrivial due to the complex interplay between the composition, structure, and local coordination environment. To address this challenge, we developed a machine-learned regression model capable of predicting Dq/*B* trained on 193 experimentally determined Dq/*B* values and their associated compositional and structural features. We then applied it to estimate the Dq/*B* values of over 6060 known inorganic structures with potential octahedral Cr^3+^ substitution sites. Eight phosphor hosts, Y_2_Mg_3_Ge_3_O_12_, YInGe_2_O_7_, LiInW_2_O_6_, Gd_3_SbO_7_, Ba_2_ScTaO_6_, Ba_2_MgWO_6_, LiLaMgWO_6_, and Ca_3_MgSi_2_O_8_, representing a range of crystal field environments were selected from this list for synthesis and characterization. Their measured Dq/*B* values closely match model predictions, demonstrating the utility of this machine-learning framework for accelerating the discovery of application-specific Cr^3+^-substituted NIR phosphors.

## Graphical abstract

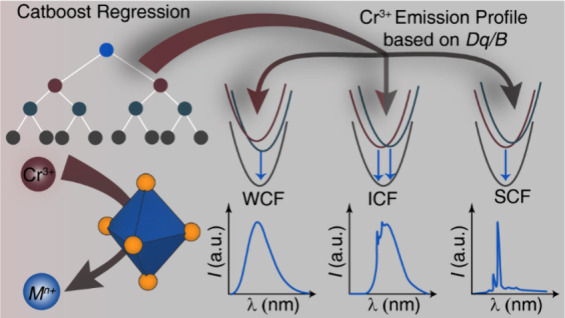

## 1 Introduction

Near-infrared (NIR) light covers three distinct regions of the electromagnetic spectrum with wavelengths ranging between 650 and 950 nm (NIR I), 1000 and 1350 nm (NIR II), and 1550 and 1870 nm (NIR III). Each region has unique applications in spectroscopy among other technologies.^1^ The NIR I region is particularly interesting because it plays a crucial role in medical diagnosis, night vision, plant cultivation, remote sensing, food quality analysis and testing, and solar cells.^2–7^ This energy window also covers the stretching vibration of C–H, O–H, and N–H bonds, enabling fast, nondestructive detection of biomolecules like sugar, protein, fat, or harmful substances such as pesticides.^8–11^ Additionally, the NIR I region is known as the first biological window because it penetrates biological tissues, making it ideal for radioisotope-free imaging and noninvasive sensing.^12^ Ultimately, NIR I photons can be detected using low-cost silicon-based detectors, making sensors operating in this region cost-effective and easy to implement.^13^

The main challenge hindering the broader application of this technology is the limited access to cheap, efficiently generated NIR I light sources.^14^ Traditional NIR sources such as incandescent bulbs, halogen lamps, and direct-emitting LEDs like GaAs/GaAlAs are all functional. However, they are generally inefficient at producing NIR photons or only have narrow (sharp line) emissions, limiting their application space. Alternatively, phosphor-converted NIR LEDs (pc-NIR LEDs) have emerged as a viable NIR I light source due to their high output power, efficiency, durability, compact size, and potential to generate narrow or broad emission depending on the phosphor used.^15–17^ Generally, NIR I producing phosphors involve introducing transition-metal ion activators such as Mn^2+^, Ni^2+^, Mn^4+^, Fe^3+^, or Cr^3+^, into suitable inorganic host materials.^18,19^ The 3*d↔*3*d* electronic transitions for each of these ions occur by absorbing visible light and down-converting the photons to various regions in the NIR; yet, most activator-host combinations fail to meet other key optical property requirements for practical applications, including efficient excitation by an LED, high conversion efficiencies (i.e., quantum yields), and controllable and variable emission peak shapes and positions. Cr^3+^-substituted inorganic phosphors are the one consistent exception, allowing them to emerge as a promising platform for generating cost-effective NIR I light. These systems are particularly desirable because Cr^3+^ can exhibit absorption in the visible region, matching the ∼ 450 nm output of widely used InGaN blue LED chips. This enables efficient blue-to-NIR down-conversion using inexpensive, readily available LEDs as the excitation source.

Indeed, Cr^3+^-based pc-NIR LEDs have been demonstrated to emit across the entire NIR I region (700–1100 nm).^17,20,21^ The variation in the emission characteristics stems entirely from differences in the Cr^3+^ 3*d↔*3*d* electronic transition, which can be fundamentally understood and controlled by tuning the interactions between Cr^3+^ and the surrounding ligands (anions) within a host crystal structure.^22^ When the Cr^3+^ is substituted on a crystallographic site with octahedral (*O*_*h*_) symmetry, the surrounding anions generate an electrostatic field, causing the nominally 5-fold-degenerate 3*d*-orbitals to split into two sets: the triply degenerate *t*_2g_ orbitals and the doubly degenerate *e*_g_ orbitals. The magnitude of this crystal field splitting energy (10*Dq*) stabilizes these orbital manifolds to different extents, depending on the host crystal structure, thereby altering the optical properties.^23,24^

This behavior is best illustrated using Tanabe-Sugano diagrams, which depict the relative energies of electronic states as a function of crystal field splitting and Racah parameters for octahedrally (or tetrahedrally) coordinated transition metal ions. In the case of octahedral Cr^3+^ in weak crystal fields (WCF) where *Dq/B* is <2.3, the emission originates from the spin-allowed ^4^T_2_ → ^4^A_2_ transition, leading to a red-shifted NIR I emission with a broad full-width-at-half-maximum (fwhm) often between 130 and 340 nm (≈1000 cm^–1^ to ≈6000 cm^–1^).^25^ Many Cr^3+^substituted phosphors have been reported with a broadband NIR I emission, such as Gd_3_Sc_2_Ga_3_O_12_:Cr^3+^ (fwhm = 1855 cm^–1^, λ_em_ = 756 nm) and Ca_3_Sc_2_Si_3_O_12_:Cr^3+^ (fwhm = 1371 cm^–1^, λ_em_ = 783 nm).^20,21^ Conversely, in strong crystal fields (SCF), where *Dq/B* is >2.3, the ^2^E state becomes the lowest energy excited state, and emission occurs from the spin-forbidden ^2^E → ^4^A_2_ transition, resulting in a comparably blue-shifted narrow-band emission. This is the case for Cr^3+^ in α-Al_2_O_3_, which results in the sharp, narrow emission lines centered between ∼ 690 nm and ∼ 720 nm that are useful for ruby lasers.^26^ These narrow emissions are also ideal for precise sensing applications or controlled environments requiring wavelength specificity. In intermediate crystal fields (ICF), where the *Dq/B* ratio is approximately 2.3, the ^4^T_2_ and ^2^E levels intersect at the minimum of their potential energy surfaces, leading to orbital mixing that produces a combination of narrow and broadband emissions. This makes these materials particularly useful for applications such as luminescence thermometry.^18,27–30^ Thus, predicting the *Dq/B* ratio based on crystal-chemical knowledge of the Cr^3+^—host interaction will enable more efficient discovery of Cr^3+^ phosphors with specific *Dq/B* for desired application spaces.

Here, we develop a machine learning model using the CatBoost regression algorithm to predict *Dq/B* for Cr^3+^-substituted phosphors. We created a training set containing 193 experimentally reported *Dq/B* values that stem from Cr^3+^-substituted phosphors, each featuring a single crystallographically independent octahedral coordination environment, sourced from peer-reviewed literature. The features were engineered using a series of compositional-related descriptors and structural features like polyhedral geometry, ionic radius of substitution cation, space group, with feature reduction revealing the dominant material properties that influence crystal field splitting.^31^ The final trained model was then applied to predict *Dq/B* values for over 6060 oxide and fluoride compositions. This was followed by experimental validation involving selecting eight compounds from this list that could be prepared by solid state synthesis. The true *Dq/B* was then determined from the absorption spectrum collected using diffuse reflectance spectroscopy. This predictive approach provides a powerful tool for identifying Cr^3+^ substituted phosphors by estimating their *Dq/B* before experimental synthesis, accelerating material discovery and development.

## 2 Methods

The machine learning model was developed using the CatBoost regression algorithm. The model validation was carried out using 10-fold cross-validation to assess predictive performance initialized with 10 different random states.^32^ The initial feature set used to build the model is provided in Table S2. Following initial evaluation, hyperparameter tuning was conducted through an exhaustive grid search approach with 10-fold cross-validation, utilizing the Scikit-Learn Python library to examine a wide range of possible parameter combinations.^33^ The search space included iterations in the range of [250, 500, 750, 1000, 1500], learning rate values of [0.08, 0.09, 0.0925, 0.095, 0.1, 0.15], depth values of [3, 4, 5, 6], l2 leaf regularization values of [1.5, 1.75, 1.9, 2.0, 2.25, 2.5], and border count values of [32, 64, 128]. The feature set was then pruned based on feature importance scoring derived internally using the CatBoost model. Once the optimal set of features was identified, hyperparameters were adjusted one final time, yielding optimal settings of iterations = 500, learning rate = 0.092, depth = 4, L2 leaf regularization = 1.9, and border count = 64. This model was used to predict the *Dq/B* of 6060 oxide and fluoride compounds featuring octahedral crystallographic sites suitable for Cr^3^^+^ substitution and having a calculated electronic bandgap >1.77 eV, as noted in the results and discussion.

The Cr^3+^- substituted phosphors suggested by the machine learning-based predictions were prepared via solid-state reactions in alumina crucibles starting from Li_2_CO_3_ (Alfa Aesar, 99.998%), La_2_O_3_ (Alfa Aesar, 99.999%), MgO (Sigma-Aldrich, 99.995%), WO_3_ (Alfa Aesar, 99.8%), Cr_2_O_3_ (Alfa Aesar, 99%), Y_2_O_3_ (Alfa Aesar, 99.9%), In_2_O_3_ (Alfa Aesar, 99%), Gd_2_O_3_ (Alfa Aesar, 99.9%), BaCO_3_ (Johnson Mathey, 99.99%), Ta_2_O_5_ (Alfa Aesar, 99.993%) CaCO_3_ (Alfa Aesar, 99%), SiO_2_ (Sigma-Aldrich, 99.5%), BaF_2_ (sigma-Aldrich, 99.5%), H_3_BO_3_ (Sigma-Aldrich, 99.5%), Sb_2_O_5_ (Alfa Aesar, 99%), and GeO_2_ (Sigma-Aldrich, 99.99%). The starting materials for each compound were loaded in the requisite stoichiometric ratios and thoroughly ground using an agate mortar and pestle. Y_2_Mg_3–3x_Cr_3*x*_Ge_3_O_12_ (*x* = 0, 0.01) were reacted at 1400 °C for 8 h. The YIn_1–*x*_Cr_*x*_Ge_2_O_7_ (*x* = 0, 0.01) system was reacted at 1250 °C for 10 h with 7.5% wt. excess Ge_2_O_3_ to compensate for evaporation during synthesis. LiIn_1–*x*_Cr_*x*_W_2_O_8_ (*x* = 0, 0.01) starting powders were decomposed at 700 °C for 5 h and then reground before reacting at 900 °C for 20 h. Gd_3_Sb_1–*x*_Cr_*x*_O_7_ (*x* = 0, 0.01) system was reacted at 1400 °C for 8h. The Ba_2_Sc_1–*x*_Cr_*x*_TaO_6_ (*x* = 0, 0.01) system reacted at 1250 °C for 6 h with 2 wt % H_3_BO_3_ as flux. Ba_2_Mg_1–*x*_Cr_*x*_WO_6_ (*x* = 0, 0.01) system was reacted at 1300 °C for 5 h. The LiLaMg_1–*x*_Cr_*x*_WO_6_ (*x* = 0, 0.01) system was reacted at 1250 °C for 8 h with 15% wt. excess Li_2_CO_3_ to compensate for evaporation during synthesis. Ca_3_Mg_1–*x*_Cr_*x*_Si_2_O_8_ (*x* = 0, 0.01) starting powders were decomposed at 700 °C for 5 h and then reground before reacting at 1400 °C for 6 h with 5% BaF_2_ as flux. All reactions mentioned above were carried out in air with furnace heating and cooling rates of 3 °C/min. The samples were checked for phase purity using powder X-ray diffraction on a PANalytical Empyrean powder diffractometer equipped with Cu Kα radiation (λ = 1.54183 Å). The lattice parameters were refined based on the Le Bail method using the GSAS package with a shifted Chebyshev function employed to model the background.^34,35^ Diffuse reflectance spectra were collected on a UV–vis spectrophotometer (JASCO V-770) with an integrated sphere attachment and BaSO_4_ powder as a standard.

## 3 Results and Discussion

### 3.1 Data Extraction and Feature Engineering

The development of the machine learning model for predicting the *Dq/B* ratio began with collecting the experimentally reported *Dq/B* values for Cr^3+^ phosphors published in the literature. The selection of phosphors was limited to systems containing only one crystallographically independent octahedral substitution site to ensure reliable calculation of *Dq/B*. The metadata associated with every report was also extracted, including compositional information, the Cr^3^^+^ ion concentrations (*x*), basic crystal structure information, and the more detailed crystallographic information file was also acquired. The final data set comprised 193 compounds, including 162 oxides and 31 fluorides. Although the data set was small, it includes a wide range of reported *Dq/B* values. The lowest value reported was 1.44 from In­(PO_3_)_3_:0.04% Cr^3+^, whereas the highest value was 3.43 for SrGa_12_O_19_:0.1% Cr^3+^.^43,44^ The type of crystal field splitting (i.e., WCF, ICF, and SCF) was then categorized for each *Dq/B* value based on the Tanabe–Sugano diagram, as illustrated in Figure 1a. The classification thresholds were defined as WCF < 2.23, 2.23 ≤ ICF ≤ 2.42, and SCF > 2.42. These cutoff values were determined through careful analysis of the Tanabe–Sugano diagram for Cr^3^^+^ (a *d* ^3^ ion), and further validated by examining the corresponding emission spectra of the phosphors to ensure consistency between theoretical predictions and experimental observations. For example, Gd_3_Ga_5_O_12_:0.02% Cr^3+^ has a *Dq/B* value of 2.43, while Ca_3_Sc_2_Si_3_O^12^: 0.03% Cr^3+^ exhibits a *Dq/B* of 2.22, highlighting the range of *Dq/B* values within the ICF reported Cr^3+^ phosphor.^45,46^ Based on these values, out of the compounds identified in the literature, 96 compounds contain Cr^3+^ in WCF spitting, 40 have ICF spitting, and 57 are SCF spitting. The training data histogram depicted in Figure 1b clearly illustrates the data imbalance, indicating that most compositions are centered around *Dq/B ≈* 2.1 to 2.2.

**Figure 1:**
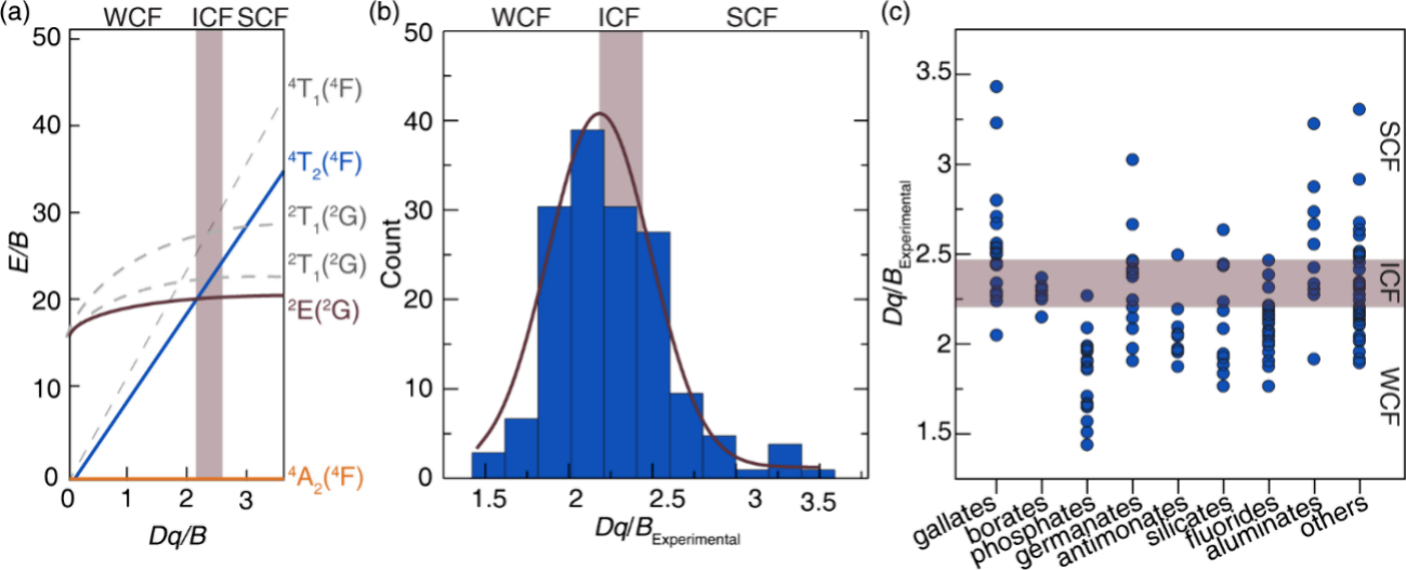
(a) Tanabe–Sugano diagram of a *d* *^3^* configuration, illustrating energy levels of electronic transitions as a function of *Dq/B*. (b) Distribution of *Dq/B* of 193 training labels. The red curve shows the trend. (c) Distribution of *Dq/B* in different crystal hosts. The shaded portion highlights the training labels of intermediate crystal field (ICF), and the regions below and above correspond to weak crystal field (WCF) and strong crystal field (SCF) training labels, respectively.

The distribution of *Dq/B* training data and the associated WCF, ICF, and SCF classifications were further analyzed by compositional class, offering insight into how specific host chemistries influence the crystal field environment. Clear trends emerge from this compositional breakdown as plotted in Figure 1c. For example, phosphates tend to fall within the weak crystal field regime, consistent with their more ionic bonding character and lower crystal field strength. In contrast, borates frequently exhibit intermediate crystal field behavior, likely due to the balance between their structural rigidity and moderately covalent bonding interactions. Aluminates often fall into the intermediate or strong field regimes, depending on factors like the bond covalency, site symmetry, and potential for octahedral distortion within the crystal structure. This simple analysis not only deepens our understanding of how host chemistry governs crystal field effects in Cr^3+^-activated phosphors, but also introduces a useful internal consistency check for model predictions. For instance, encountering a phosphate host categorized within the strong crystal field regime would be highly unusual. It may signal an anomaly in the crystal field assignment or an issue with the underlying data. Thus, compositional trends serve both as a scientific guide and a diagnostic tool for assessing the reliability of *Dq/B* classifications within diverse host environments. The complete set of training labels is detailed in Table S1 of the Supporting Information.

Once the data set was constructed, a feature set was engineered specifically to represent *Dq/B* in each host structure. This process involved expanding 35 distinct compositional (elemental) variables through five mathematical operations: weighted average, difference, maximum, minimum, and standard deviation, where the weights were determined by the stoichiometric coefficients of the elements in the host composition.^36^ The resulting 175 compositional features represent the relative positions of atoms in the periodic table, their electronic configurations, and their physical characteristics.^37^ For instance, atomic number, atomic weight, Mendeleev’s number, and covalent radius were included to account for differences in size, mass, and chemical similarity among atoms.^38^ Electron affinity and electronegativity were considered to model electronic excitation energies and chemical trends across various materials. The compositional feature set was further expanded by incorporating 26 structural features encompassing attributes such as the host crystal system, space group, point group, polyhedral volume of the substitution site, and radius of the substituting cation. Additionally, the reported concentration of Cr^3^^+^ (*x*) was included, as was a feature defined as 1/*R* ^2^, where R = |avg. metal—anion bond length| to emphasize the electrostatic interactions between the substituting metal cation and the anions within the polyhedron. A total of 203 features were generated before feature reduction was employed to build a statistically robust model.

### 3.2 Model Training

A regression-based machine learning approach was employed to uncover structure–property relationships of known Cr^3+^ phosphors and enable prediction of *Dq/B* values for undiscovered Cr^3+^-substituted phosphors. The CatBoost regression model was selected particularly for its efficiency with small data sets.^32^ It uses decision trees as weak learners and enhances their performance through boosting, an iterative process where each tree corrects the errors of the previous one.^32^ The data were standardized to achieve a mean of 0 and a variance of 1, with the initial model hyperparameters optimized using an exhaustive grid search, including the number of iterations, learning rate, maximum depth, L2 leaf regularization, and border count as described in the experimental section. The importance of each feature was then determined using CatBoost’s built-in feature importance score method, which quantifies feature contributions by calculating the average decrease in prediction error when each feature is included. Specifically, CatBoost assesses the significance of features based on how frequently and effectively they reduce the prediction error across all decision trees within the ensemble. The complete list of initial features with their importance score is provided in Table S2.

Feature reduction was performed by iteratively removing the least important features based on these scores. The selection process began with the top 5 features, and R^2^-scores were evaluated using 10-fold cross-validation. This evaluation was repeated at intervals of 5 features, increasing the feature set incrementally from 5 to all 203 features. Figure S1 illustrates the model’s performance as a function of the number of selected features. The final analysis revealed that the highest performance was achieved using 15 features, giving an average *k*-fold cross-validated R^2^-score of 0.73. The final 15 reduced features and their associated importance scores are shown in Figure 2a. Figure S2, which presents the correlation matrix of the selected features, clearly illustrates that the majority of these features are independent, signifying minimal redundancy among the chosen descriptors and emphasizing their unique contributions to the model’s predictive efficacy.

**Figure 2:**
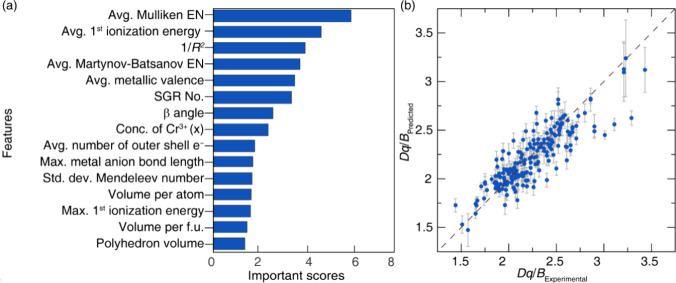
(a) Feature importance ranking for predicting *Dq*/*B* values in Cr^3^^+^-substituted phosphors, derived from the trained regression model. The most influential features include the average Mulliken electronegativity, average first ionization energy, and the inverse squared metal–ligand distance (1/*R* ^2^), all of which relate directly to the strength of the metal–ligand interactions that govern crystal field effects. Additional important descriptors include the average Martynov-Batsanov electronegativity, average metallic valence, structural parameters (e.g., space group number and β angle), and compositional features such as the concentration of Cr^3^^+^(*x*). (b) The parity plot compares predicted versus experimental values of *Dq*/*B* show good agreement with most points clustering near the identity line (dashed line). Error bars represent model uncertainty.

Examining the feature importance reveals key chemical and structural descriptors that govern crystal field splitting strength in Cr^3+^-substituted phosphors. The host’s average Mulliken electronegativity (EN), calculated as the average EN values based on the stoichiometrically weighted composition, shows the highest importance score among all features. This highlights its central role in the material’s electron-withdrawing capability, which directly affects the electronic distribution around the Cr^3+^ ion and the strength of metal–anion interactions. Among structural descriptors, 1/*R* ^2^ (R = |avg. metal—anion bond length|) is very influential. A smaller cation radius leads to stronger electrostatic interactions and shorter bond distances, resulting in a more pronounced crystal field. The average metal–anion bond length­(*R*) also ranks highly in importance, which makes sense since it is directly related to crystal field splitting. Shorter Cr^3+^–anion distances enhance the strength of the crystal field due to increased Coulombic attraction and greater overlap between the Cr^3+^ 3*d* orbitals and anion orbitals. This contributes to higher *Dq* values and a shift toward stronger crystal field classification. Ionization energy of the host cations also plays a significant role, as it reflects the ability of surrounding atoms to polarize electron density toward the Cr^3+^ center. Higher ionization energies generally indicate more tightly held electrons, leading to stronger crystal fields through reduced shielding and enhanced bonding interactions. Finally, space group symmetry (SGR No.) is another nontrivial factor influencing *Dq/B*. It encapsulates the crystal structure’s broader geometric and symmetry constraints, which in turn dictate the potential for distortion and coordination environment of the Cr^3+^ site. Higher-symmetry environments often preserve ideal octahedral coordination, while lower-symmetry structures may induce distortions that alter orbital degeneracy and crystal field strength. Together, these features provide a physically interpretable and chemically intuitive understanding of how the local and global structure of the host structure influences the crystal field environment and offer design guidelines for engineering host structures with tailored crystal field strengths.

After selecting the 15 optimal features, a final round of hyperparameter tuning was performed to confirm the model’s robustness. This tuning is valuable because the model dynamics can change after feature selection, requiring reoptimization to maintain the best predictive performance. During this step, the model’s uncertainty for each predicted data point was also estimated using bootstrap aggregating (bagging).^39^ This statistical technique generates sample data sets from an initial data set by randomly drawing with replacement. These bootstrapped sample data sets are representative of the actual data distribution and can be used to evaluate the variance (standard deviation) of machine-learning estimators. Here, each bootstrapped subsample data set is fitted to ten parallel models. The prediction’s variance is then calculated by adding each subset together. Finally, 10 random states of the 10-fold validation were performed to ensure a robust model. The validation results and uncertainty of each prediction for the optimized model between the CatBoost predicted *Dq/B* and experimentally reported *Dq/B*, illustrated in Figure 2b, show a reasonable R^2^-score of 0.75 and mean absolute error (MAE) of 0.13 for 10-fold cross-validation. These results indicate the model’s predictive capability and reliability, even when applied to this small data set.

Examining the parity plot shows a slight underestimation for *Dq/B* values exceeding 2.75; however, this is likely due to the limited number of compounds in the training data set containing strong Cr^3^^+^ crystal fields. The error distribution (Figure S3) across the predicted compositions shows that 94% of the training data exhibit an error of less than 15% between the model and the ground truth. The overall data set does tend to skew toward underestimation on average. Further breaking down the training data based on the type of crystal field splitting shows that the predicted *Dq/B* is MAE is 0.118 for WCF, 0.116 for ICF, and 0.156 for SCF, indicating rather uniform error across each crystal field classification.

### 3.3 Experimental Validation through Novel Material Synthesis

This operational model was applied to predict the *Dq/B* values for 6060 reported oxides and fluorides from the Materials Project database. This number is limited compared to the entire database because of a few imposed criteria. First, only oxides and fluorides were considered since these are the anions composing the training data set. Second, only compositions featuring octahedral coordination environments for Cr^3+^ to occupy were included. Additional compositional constraints were also considered, as outlined in Figure 3a. Compositions containing group 18 elements, as well as Cd, Ag, Au, Hg, Tl, Pb, S, Cl, Br, At, and hydrogen-containing compounds, were excluded from our prediction set because they are rarely reported to be suitable inorganic phosphor hosts and are not easy to synthesize. Further, only the compounds that could be synthesized under ambient pressure and are energetically favorable at room temperature (on the convex hull) were considered. Finally, the calculated electronic band gap (*E*_*g*_) for every compound was required to be greater than 1.77 eV. This threshold was chosen to ensure compatibility with broadband emission in the NIR I region, which spans wavelengths from 700 to 1100 nm. According to the photon energy-wavelength relationship (*E* = 1240/λ), a wavelength of 700 nm corresponds to a photon energy of approximately 1.77 eV. Therefore, materials with *E*_*g*_ ≥ 1.77 eV (accounting for the ∼ 50% underestimation associated with semilocal DFT) are required to host the Cr^3+^ electronic transition without reabsorption losses. The histogram of predicted *Dq/B* values for 6060 compounds are plotted in Figure 3b. Most compositions have a *Dq/B* ≈ 2.1 to 2.3, reflecting the data distribution present in the training set. The model categorized 2740 compositions as WCF, 1764 as ICF, and 1556 as SCF using the previously defined classification thresholds: WCF < 2.23, 2.23 ≤ ICF ≤ 2.42, and SCF > 2.42.

**Figure 3:**
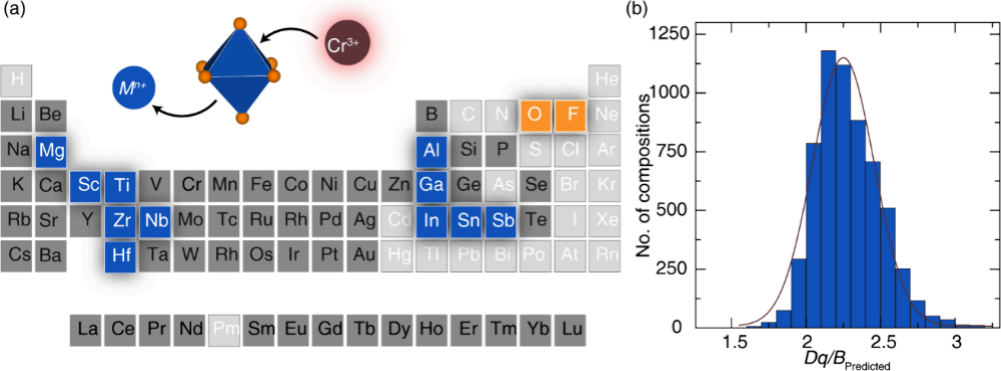
(a) The elements that compose Cr^3^^+^-substituted phosphor hosts. Anions in orange and octahedral environments centered by one of the blue highlighted atoms were included, and compositions with any species outside of the orange anions, blue substituted cations or dark gray elements were excluded from the screening set. (b) Distribution of predicted *Dq/B* of 6060 compounds in the screening set. The red curve shows the distribution.

From this list, eight different phosphor hosts were selected as the candidate materials for experimental validation. These compounds were chosen to represent all three crystal field regions: strong crystal field (SCF), intermediate crystal field (ICF), and weak crystal field (WCF). This is advantageous for assessing the model’s accuracy across the full range of possible Cr^3+^-substituted phosphors. The selected materials, based on predicted *Dq/B* and estimated uncertainty, were also intentionally chosen to represent diverse chemical compositions and crystal structures. The specific compounds identified for investigation in this study are Y_2_Mg_3_Ge_3_O_12_:1% Cr^3+^ (predicted *Dq/B* = 2.38 ± 0.02; ICF),^40^ YInGe_2_O_7_:1% Cr^3+^ (predicted *Dq/B* = 2.02 ± 0.02; WCF),^41^ LiInW_2_O_8_:1% Cr^3+^ (predicted *Dq/B* = 1.96 ± 0.05; WCF), Gd_3_SbO_7_:1% Cr^3+^ (predicted *Dq/B* = 2.28 ± 0.03; ICF),^42^ Ba_2_ScTaO_6_:1% Cr^3+^ (predicted *Dq/B* = 2.16 ± 0.04; WCF),^43^ Ba_2_MgWO_6_:1% Cr^3+^ (predicted *Dq/B* = 2.30 ± 0.05; ICF),^44^ LiLaMgWO_6_:1% Cr^3+^ (predicted *Dq/B* = 2.46 ± 0.06; SCF),^45^ and Ca_3_MgSi_2_O_8_:1% Cr^3+^ (predicted *Dq/B* = 2.32 ± 0.02; ICF).^46^

All eight compounds, illustrated in Figure 4, were then synthesized using conventional high-temperature solid-state reaction. Phase purity was determined using Le Bail refinements of laboratory powder X-ray diffraction data collected using Cu Kα radiation. The corresponding refined diffractograms are plotted in Figure S4, with refinement statistics and refined unit cell parameters summarized in Table S3. The analysis indicates the successful formation of single-phase products, with the exception of Ba_2_MgWO_6_, which has slightly impurity phase of BaWO_4_. However, BaWO_4_ lacks crystallographic sites for Cr^3+^ substitution. Thus, all eight compounds are suitable for further structural and spectroscopic (*Dq/B*) evaluation.

**Figure 4:**
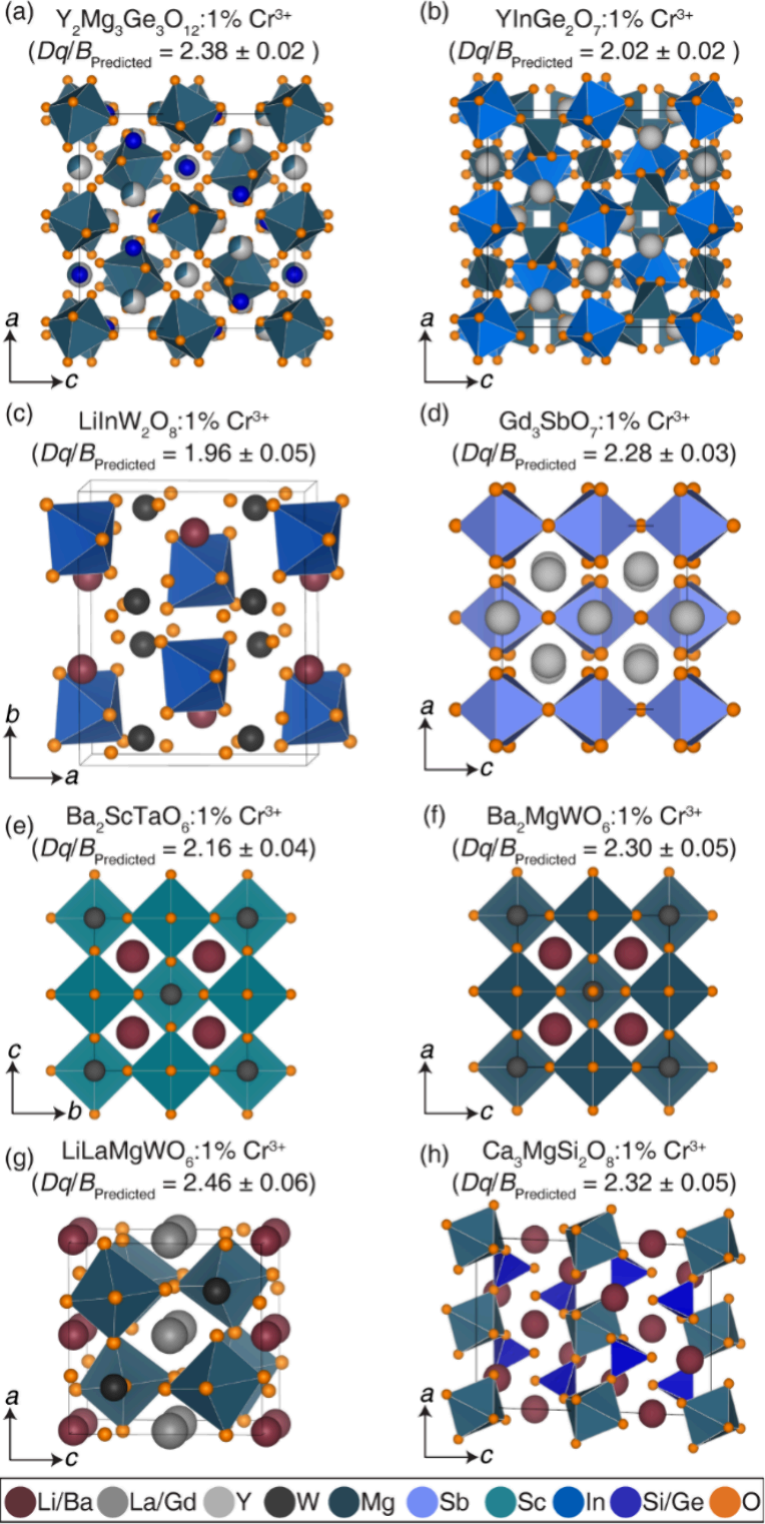
Phosphor hosts considered in the experimental verifications are (a) Y_2_Mg_3_Ge_3_O_12_:1% Cr^3+^, (b) YInGe_2_O_7_:1% Cr^3+^, (c) LiInW_2_O_8_:1% Cr^3+^, (d) Gd_3_SbO_7_:1% Cr^3+^, (e) Ba_2_ScTaO_6_:1% Cr^3+^, (f) Ba_2_MgWO_6_:1% Cr^3+^, (g) LiLaMgWO_6_:1% Cr^3+^, and (h) Ca_3_MgSi_2_O_8_:1% Cr^3+^.

The diffuse reflectance spectrum of each phosphor and its corresponding unsubstituted host structure was collected after synthesis, and the data are plotted in Figure 5. All Cr^3+^-substituted phosphors exhibit intense absorption in the blue and red regions of the visible spectrum, corresponding to the ^4^A_2_ → ^4^T_1_ and ^4^A_2_ → ^4^T_2_ of Cr^3+^ electronic transitions, respectively. Table 1 shows the wavelengths (and energies) corresponding to these transitions for each phosphor.

**Figure 5:**
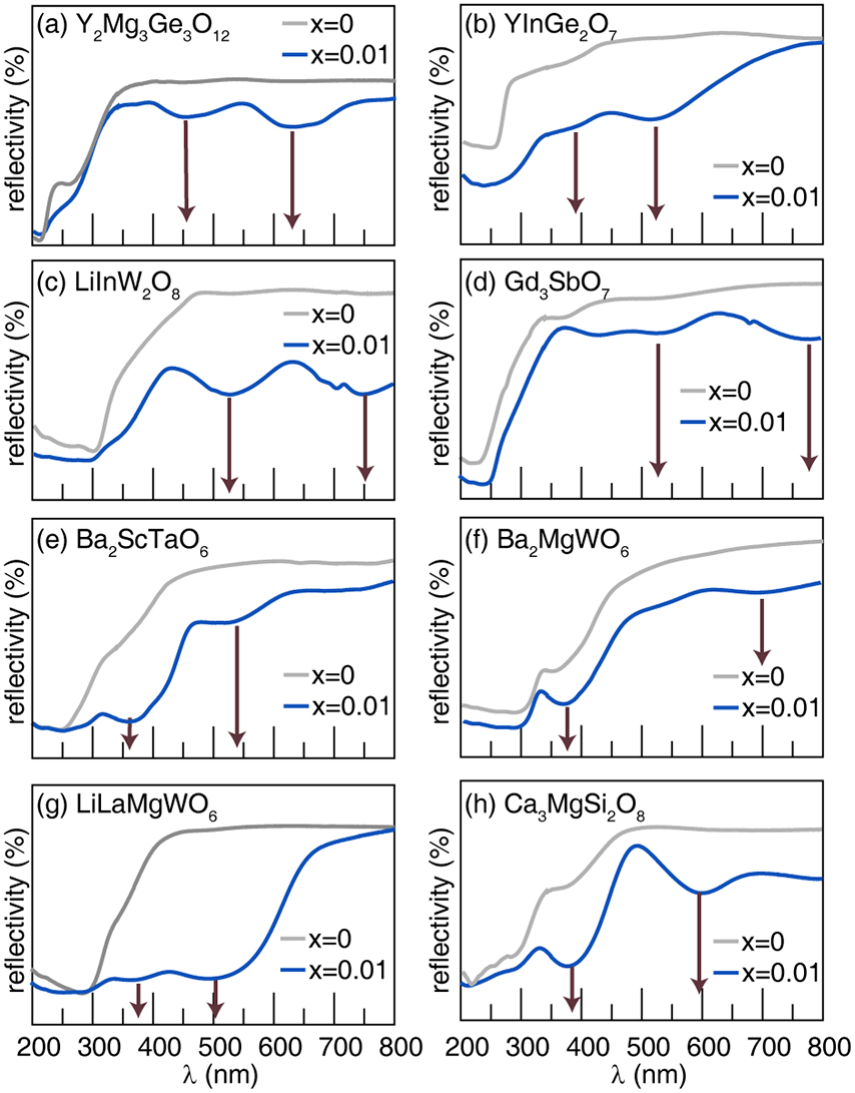
Diffuse reflectance spectra of (a) Y_2_Mg_3_Ge_3_O_12_, (b) YInGe_2_O_7_, (c) LiInW_2_O_8_, (d) Gd_3_SbO_7_, (e) Ba_2_ScTaO_6_, (f) Ba_2_MgWO_6_, (g) LiLaMgWO_6_, and (h) Ca_3_MgSi_2_O_8_ with 0% and 1% of Cr^3+^ phosphors. Solid red arrows represent the wavelength corresponding to the transitions from ^4^A_2_ to ^4^T_1_, and ^4^A_2_ to ^4^T_2_, respectively.

**Table 1:** Wavelengths (in nm and eV) Corresponding to the ^4^A_2_ → ^4^T_1_ and ^4^A_2_ → ^4^T_2_ Transitions in Each Composition and Their Calculated *Dq*, *B*, and *Dq/B*

composition	*E* **(** ^4^ **A**_**2**_ **→** ^4^ **T**_**1**_ **)**	*E* **(** ^4^ **A**_**2**_ **→** ^4^ **T**_**2**_ **)**	*Dq* (cm^–1^)	*B* (cm^–1^)	*Dq/B*
(a) Y_2_Mg_2.97_Cr_0.03_Ge_3_O_12_	449 nm/2.76 eV	633 nm/1.96 eV	1581	653	2.41
(b) YIn_0.99_Cr_0.01_Ge_2_O_7_	377 nm/3.29 eV	520 nm/2.38 eV	1923	717	2.68
(c) LiIIn_0.99_Cr_0.01_W_2_O_8_	519 nm/2.39 eV	752 nm/1.65 eV	1331	621	2.13
(d) Gd_3_Sb_0.99_Cr_0.01_O_7_	537 nm/2.30 eV	775 nm/1.60 eV	1290	595	2.17
(e) Ba_2_Sc_0.99_Cr_0.01_TaO_6_	362 nm/3.42 eV	522 nm/2.38 eV	1915	878	2.18
(f) Ba_2_Mg_0.99_Cr_0.01_WO_6_	370 nm/3.42 eV	530 nm/2.46 eV	1887	839	2.25
(g) LiLaMg_0.99_Cr_0.01_WO_6_	363 nm/3.42 eV	504 nm/2.46 eV	1984	766	2.60
(h) Ca_3_Mg_0.99_Cr_0.01_Si_2_O_8_	390 nm/3.18 eV	590 nm/2.10 eV	1695	974	1.74

The crystal field strength can be characterized by crystal field splitting (*Dq*) and the Racah parameter (B) calculated using eqs 1–3.^47^ Analysis of the data presented in Figure 5 and summarized in Table 1 yields each composition’s calculated *Dq* and *B* values. The resulting experimentally measured *Dq/B* ratios are also provided in Table 1.

10Dq=E(T42)=E(A42→T42) 1

$$\mathrm{Dq}/B=\left(15\left(x-8\right)\right)/\left(x^{2}-10x\right)$$ 2

x=(E(A24→T14)−E(A24→T24))/Dq 3

These experimental *Dq/B* values indicate the crystal field splitting of Cr^3+^ is SCF in YinGe_2_O_7_ and LiLaMgWO_6,_ WCF for Cr^3+^ in LiInW_2_O_8_, Gd_3_SbO_7_, Ba_2_ScTaO_6_, and Ca_3_MgSi_2_O_8_, and ICF for Cr^3+^ in Y_2_Mg_3_Ge_3_O_12_ and Ba_2_MgWO_6_. Comparing these experimental measurements with the predictions made by the trained model, listed in Table 2, shows excellent agreement for most compositions, with discrepancies observed in predicting the crystal field strength of Cr^3+^ in YInGe_2_O_7_, Gd_3_SbO_7_, and Ca_3_MgSi_2_O_8_. Notably, for Gd_3_SbO_7_, the predicted ICF is close in *Dq/B* value to the experimental WCF, suggesting that minor tuning of the crystal field environment could shift it into the predicted region. These results support that our machine-learning model has good predictive capability, allowing this approach to act as a dependable tool to support the search the identification of Cr^3+^ phosphors regardless of application.

**Table 2:** Comparison between the Predicted *Dq/B* and Experimental *Dq/B* Values

composition	pred. *Dq/B*	exp. *Dq/B*
(a) Y_2_Mg_2.97_Cr_0.03_Ge_3_O_12_	2.38 ± 0.02 (ICF)	2.41 (ICF)
(b) YIn_0.99_Cr_0.01_Ge_2_O_7_	2.02 ± 0.02 (WCF)	2.68 (SCF)
(c) LiIIn_0.99_Cr_0.01_W_2_O_8_	1.96 ± 0.05 (WCF)	2.13 (WCF)
(d) Gd_3_Sb_0.99_Cr_0.01_O_7_	2.28 ± 0.03 (ICF)	2.17 (WCF)
(e) Ba_2_Sc_0.99_Cr_0.01_TaO_6_	2.16 ± 0.04 (WCF)	2.18 (WCF)
(f) Ba_2_Mg_0.99_Cr_0.01_WO_6_	2.30 ± 0.05 (ICF)	2.25 (ICF)
(g) LiLaMg_0.99_Cr_0.01_WO_6_	2.46 ± 0.06 (SCF)	2.60 (SCF)
(h) Ca_3_Mg_0.99_Cr_0.01_Si_2_O_8_	2.32 ± 0.05 (ICF)	1.74 (WCF)

## 4 Conclusion

A machine learning model for predicting the *Dq/B* of Cr^3+^ in octahedral environments was developed by constructing a training data set extracted from phosphor literature. Eight compounds selected from the prediction, Y_2_Mg_3_Ge_3_O_12_, YInGe_2_O_7_, LiInW_2_O_8_, Gd_3_SbO_7_, Ba_2_ScTaO_6_, Ba_2_MgWO_6_, LiLaMgWO_6_, and Ca_3_MgSi_2_O_8_ were synthesized with 1% Cr^3+^ substitution, followed by optical characterization. The experimentally calculated *Dq/B* ratio confirmed Cr^3+^ presence in WCF for Cr^3+^ in LiInW_2_O_8_, Gd_3_SbO_7_, and Ba_2_ScTaO_6_, ICF for Cr^3+^ in Y_2_Mg_3_Ge_3_O_12_ and Ba_2_MgWO_6_, and SCF for Cr^3+^ in LiLaMgWO_6_, aligning with our model’s predictions. Discrepancies were observed for YInGe_2_O_7_, Gd_3_SbO_7_, and Ca_3_MgSi_2_O_8_; however, in the case of Gd_3_SbO_7_, the experimental *Dq/B* value of 2.18 lies just below the ICF range (2.23 ≤ ICF ≤ 2.42), indicating that the model still captured the underlying crystal field strength and slight tuning of the host environment around Cr^3+^ could shift this composition into the predicted region. The feature importance analysis indicated that host-related chemical properties, including average Mulliken electronegativity, first ionization energy, and 1/*R* ^2^ (R = |avg. metal–anion bond length|), are vital descriptors playing crucial role in influencing crystal field strength of Cr^3+^. These results highlight the power of data science approaches for advancing materials discovery, including cases where only small training data sets are available – provided appropriate models are employed. This framework can be extended beyond *Dq/B* to predict additional optical properties of Cr^3+^, including emission or excitation wavelengths of Cr^3+^, thereby enhancing its applicability in phosphor design.

## Supplementary Material



## Data Availability

The training and test data sets, prediction data set of 6060 compounds, as well as the codes used to generate the models in this work are openly available in https://github.com/BrgochGroup/DqBpredictor, ref 48.

## Notes

Coefficient of determination (R^2^) vs number of features (Figure S1), correlation matrix of input features (Figure S2), error distribution between predicted and measured Dq/B (Figure S3), Le Bail refinements of synthesized compounds (Figure S4), training labels and Cr^3^^+^ doping concentrations (Table S1), feature importance scores (Table S2), and refinement statistics and unit cell parameters (Table S3) (PDF)
