# Supplementary material for: Machine-Learning-Assisted Discovery of Cr3+-Based Near-Infrared Phosphors
Source: Chem Mater. 2025 Sep 19;37(19):7762–70. doi: 10.1021/acs.chemmater.5c01208 (PMC12529912; doi:10.1021/acs.chemmater.5c01208)
Supplement: Supplementary file 1 [file cm5c01208_si_001.pdf]

Machine Learning-Assisted Discovery of Cr^{3+}-based Near-Infrared Phosphors

Amit Kumar[1,2], Arslan Akbar[1,2], Hannah Lesmes[1,2], Seán R. Kavanagh[3], David O. Scanlon[4,*], and Jakoah Brgoch[1,2,*]

[1]*Department of Chemistry, University of Houston, Houston, Texas, 77204, United States.*

[2]*Texas Center for Superconductivity, University of Houston, Houston, Texas, 77204, United States.*

[3]*Harvard University Center for the Environment, Cambridge, Massachusetts, 02138, United States.*

[4]*School of Chemistry, University of Birmingham, Birmingham, B15 2TT, United Kingdom.*

**Corresponding authors*

Email: jbrgoch@uh.edu, d.o.scanlon@bham.ac.uk

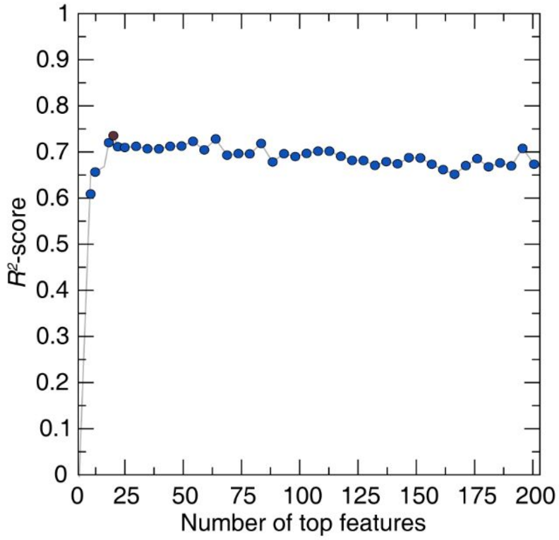

Figure S1. Coefficient of determination (R^2- score) as a function of the number of features. The red dot is showing the best R^2-score.

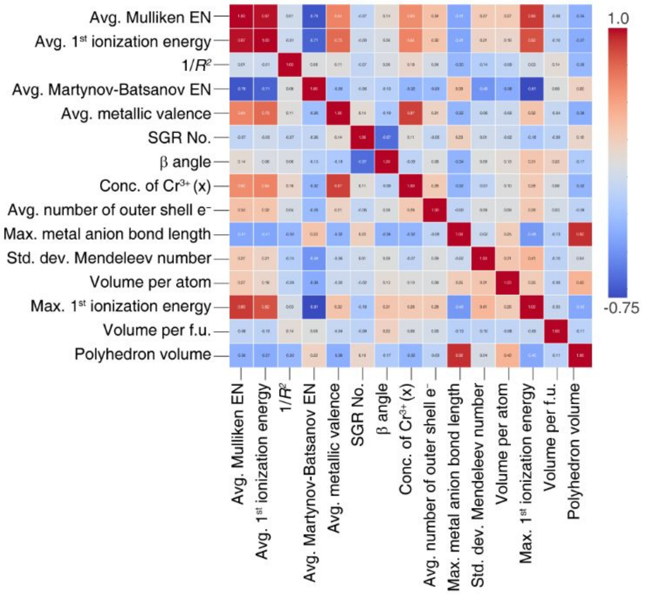

Figure S2. Correlation matrix of all input features used in the regression model. The color scale indicates the degree of linear correlation, with red representing strong positive correlation and blue representing strong negative correlation. Values in each cell denote the correlation coefficient between corresponding feature pairs.

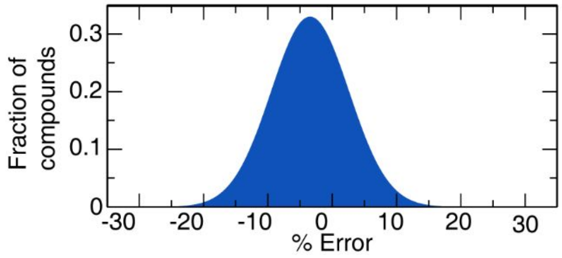

Figure S3. Fraction of compounds according to their percent error between predicted Dq/B and measured Dq/B. The red curve shows the trend

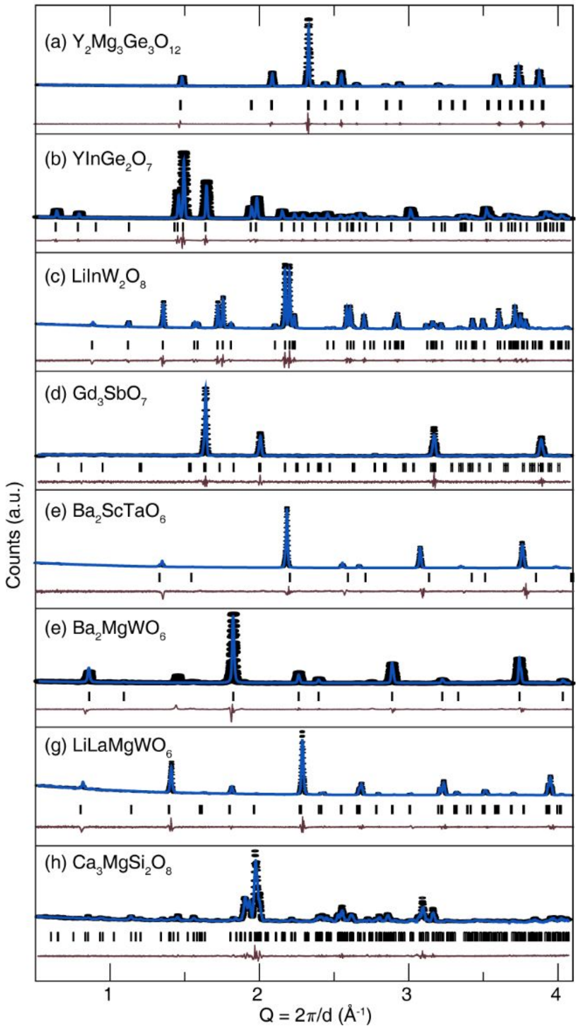

Figure S4. Le Bail refinements of (a) $Y_2Mg_3Ge_3O_{12}$, (b) $YInGe_2O_7$, (c) $LiInW_2O_8$, (d) Gd_3SbO_7, (e) Ba_2ScTaO_6, (f) Ba_2MgWO_6, (g) $LiLaMgWO_6$, and (h) $Ca_3MgSi_2O_8$ using X-ray powder diffraction data. The measured data are shown in black, the fit by the blue line, and the difference between the data and the fit by the red line.

Table S1. Training labels and Cr^{3+} doping concentrations (x).

**Formula**	***Dq/B***	**x**	**Formula**	***Dq/B***	**x**	**Formula**	***Dq/B***	**x**
Cs_2KGaF_6	1.75	0.1	Cs_2NaScF_6	1.86	0.01	$Sc_2(WO_4)_3$	2.23	0.06
K_3ScF_6	2.09	0.15	Cs_2NaGaF_6	2.19	0.2	$Sc_2(MoO_4)_3$	2.28	0.02

Rb_2NaScF_6	1.98	0.03	K_2NaInF_6	2.17	0.25	Cs_2NaAlF_6	2.14	0.01
K_2NaScF_6	2.06	0.1	K_2NaScF_6	2.06	0.1	K_3GaF_6	1.89	0.03
$BaZr(GeO_3)_3$	2.19	0.01	$KInP_2O_7$	1.65	0.05	K_3AlF_6	1.94	0.03
$BaZr(SiO_3)_3$	2.17	0.005	$Sc(PO_3)_3$	1.86	0.05	Ba_2NbInO_6	2.0	0.01
$BaHf(SiO_3)_3$	1.91	0.03	Sr_2YSbO_6	1.86	0.03	Ba_2TaInO_6	2.4	0.01
K_2LiScF_6	2.05	0.03	Ba_2ScNbO_6	2.12	0.01	$BaSi_3SnO_9$	2.01	0.01
$KScP_2O_7$	1.67	0.05	Sr_2TaInO_6	1.9	0.01	Sr_2NbInO_6	2.09	0.01
$BaIn_2(P_2O_7)_2$	1.91	0.02	Sr_2InSbO_6	1.96	0.03	$KInP_2O_7$	1.65	0.05
$SrLiGaF_6$	2.2	0.04	Ca_2NbInO_6	2.16	0.01	$Mg_2Al_4Si_5O_{18}$	2.43	0.02
$Sr_3Sc_4O_9$	2.61	0.02	$La_3Sc_2(GaO_4)_3$	2.27	0.01	Ba_2ScSbO_6	2.08	0.03
$Ca_3Y_2(GeO_4)_3$	2.13	0.01	$SrHfO_3$	2.18	0.005	$K_2Sr(Ge_4O_9)_2$	2.23	0.1
Sr_2ScSbO_6	2.04	0.03	$Na_3Li_3In_2F_{12}$	1.98	0.04	Sr_2ScSbO_6	2.03	0.03
$LaScO_3$	2.67	0.001	$LiLa_2SbO_6$	1.94	0.01	La_2MgZrO_6	2.49	0.02
$La_3Ga_5SiO_{14}$	2.62	0.1	$LiInP_2O_7$	2.09	0.04	$In(PO_3)_3$	1.44	0.04
$Ga_4Bi_2O_9$	2.31	0.008	$Ga_4Bi_2O_9$	2.31	0.03	Ca_2YSbO_6	1.95	0.03
$Al(PO_3)_3$	1.66	0.04	$Gd_3Sc_2(GaO_4)_3$	2.45	0.01	$Na_3Li_3Sc_2F_{12}$	2.06	0.07
$NaSc(GeO_3)_2$	1.96	0.03	$NaSc(GeO_3)_2$	1.96	0.03	$Gd_3Sc_2(GaO_4)_3$	2.45	0.01
Ca_2TaInO_6	2.26	0.02	$Y_3In_2(GaO_4)_3$	2.24	0.02	$KAlP_2O_7$	1.9	0.04
$KGaP_2O_7$	1.71	0.05	K_2LiAlF_6	2.3	0.01	$KGaP_2O_7$	1.96	0.08
$NaInP_2O_7$	1.97	0.04	$CaHfO_3$	2.59	0.002	$Ca_3Sc_2(GeO_4)_3$	2.65	0.14
Ca_2ScNbO_6	2.03	0.01	$GdScO_3$	2.71	0.005	$NaIn(GeO_3)_2$	1.89	0.02
$SrSc_2O_4$	1.95	0.01	$Y_3Sc_2(GaO_4)_3$	2.52	0.01	Ca_2ScSbO_6	2.18	0.03
$SrLiAlF_6$	2.05	0.05	Sr_2MgWO_6	2.9	0.001	Ca_2ScTaO_6	1.94	0.02
Sr_2TaAlO_6	2.41	0.006	In_2TeO_6	2.15	0.01	Sr_2TaAlO_6	2.41	0.006
$Gd_3Ga_5O_{12}$	2.53	0.01	$Gd_3Ga_5O_{12}$	2.44	0.02	Ga_5GdO_{12}	2.44	0.02
Sr_2TaGaO_6	2.34	0.006	Zn_4InGaO_7	2.05	0.01	Sr_2TaGaO_6	2.34	0.006
$LiSc(GeO_3)_2$	1.89	0.07	$LaGaO_3$	2.8	0.005	$NbAlO_4$	2.32	0.01
$LiCaAlF_6$	2.15	0.05	La_2MgGeO_6	3.01	0.003	$NaIn(SiO_3)_2$	1.93	0.04
Ca_2MgWO_6	2.48	0.004	$Y_3Sc_2Al_3O_{12}$	2.26	0.05	$CaScAlSiO_6$	2.07	0.01
$Al(PO_3)_3$	1.57	0.04	$ScNbO_4$	1.88	0.015	$Ca_3Ga_2(GeO_4)_3$	2.4	0.01
$NaSc(SiO_3)_2$	2.07	0.04	$NaSc(SiO_3)_2$	1.82	0.06	$CaSc_2O_4$	2.1	0.01
$NaGaP_2O_7$	1.98	0.06	$Ga(PO_3)_3$	1.51	0.04	$Ca_3Sc_2(SiO_4)_3$	2.22	0.03
$LiIn(SiO_3)_2$	1.75	0.06	Mg_2SnO_4	2.66	0.001	$CaGdAlO_4$	2.72	0.003
$Gd_2Mg_3(GeO_4)_3$	2.64	0.06	$Na_3Li_3Ga_2F_{12}$	2.1	0.05	Zn_2SnO_4	2.3	0.002
$Y_3Ga_5O_{12}$	2.53	0.02	$Y_3Ga_5O_{12}$	2.53	0.02	$Gd_3Al_2Ga_3O_{12}$	2.65	0.04
$Lu_3Ga_5O_{12}$	2.51	0.08	$Lu_3Ga_5O_{12}$	2.67	0.01	$Gd_3Al_4GaO_{12}$	2.73	0.12
$LiInF_4$	2.0	0.02	$LiInSiO_4$	2.42	0.015	Ca_2NbGaO_6	2.54	0.004
$Ca_3Sc_2(SiO_4)_3$	2.22	0.03	$MgTa_2O_6$	2.5	0.021	$LiSc(SiO_3)_2$	1.87	0.1
Na_3AlF_6	1.98	0.01	$LiGa(WO_4)_2$	2.19	0.005	$NaAlP_2O_7$	1.99	0.04
$BaAl_4(SbO_6)_2$	2.48	0.025	$MgGeO_3$	2.07	0.25	$LiGaP_2O_7$	1.87	0.12
$MgGeO_3$	2.07	0.25	Ca_2NbAlO_6	2.65	0.003	$Al_6Ge_2O_{13}$	1.87	0.03
$LaSc_3(BO_3)_4$	2.05	0.023	$TaGaO_4$	2.29	0.006	Ga_4GeO_8	2.16	0.02
$LiInO_2$	2.14	0.01	$Mg_4Nb_2O_9$	2.47	0.005	$Mg_4Sb_2O_9$	1.95	0.02
$Y_3Al_5O_{12}$	2.86	0.02	$Y_3Al_5O_{12}$	2.86	0.02	$SrGa_{12}O_{19}$	3.23	0.07
$SrGa_{12}O_{19}$	3.43	0.1	$Mg_4Ta_2O_9$	2.1	0.03	$Na_3Li_3Al_2F_{12}$	2.27	0.05
$Li_2Ti_3ZnO_8$	3.29	0.008	$CaLu_2Mg_2(SiO_4)_3$	2.57	0.05	$BaMgAl_{10}O_{17}$	2.52	0.05
$Mg_4Ga_4Ge_3O_{16}$	2.37	0.005	$Lu_3Al_5O_{12}$	2.54	0.08	$BaAl_{10}ZnO_{17}$	2.52	0.003
$LiMgGaF_6$_Mg	2.02	0.11	$LiMgGaF_6$_Ga	2.14	0.11	$LiMgAlF_6$	2.03	0.05
$Mg(GaO_2)_2$	2.54	0.008	$Zn(GaO_2)_2$	2.71	0.008	Li_2MgZrO_4	2.26	0.008
$LiScO_2$	2.37	0.03	$Y_4Al_4O_{12}$	3.21	0.02	$YAlO_3$	3.21	0.02
$Mg_7Ga_2GeO_{12}$	2.39	0.01	$ScBO_3$	2.15	0.02	$LiGa_5O_8$	2.56	0.003

$LaMgAl_{11}O_{19}$	2.9	0.02	$Mg_2B_2O_5$	2.27	0.003	$MgAl_2O_4$	2.29	0.025
Al_2ZnO_4	2.62	0.008	$GdAl_3(BO_3)_4$	2.3	0.01	$GdAl_3(BO_3)_4$	2.3	0.12
$YAl_3(BO_3)_4$	2.37	0.01	$YAl_3(BO_3)_4$	2.32	0.12	MgO	2.4	0.03
$MgY_2Al_4SiO_{12}$	3.11	0.02	$Y_3Al_2Ga_3O_{12}$	2.57	0.02	$InTaO_4$	1.75	0.04
$CaMgGe_2O_6$	2.12	0.02	$NaScSi_2O_6$	2.07	0.04	K_2NaGaF_6	2.23	0.01
$Lu_2CaMg_2Si_3O_{12}$	2.67	0.05	Ga_2O_3	2.56	0.01	$NaScGe_2O_6$	1.96	0.03
K_2LiAlF_6	1.87	0.07	K_2LiGaF_6	1.85	0.07	K_2LiInF_6	1.77	0.07
$LiInGe_2O_6$	2.03	0.08	Sr_2GaSbO_6	2.26	0.03	$Mg_2Al_4Si_5O_{18}$	2.43	0.02
$Y_2Mg_2Ga_2Si_2O_{12}$	2.45	0.04	Sr_2GaTaO_6	2.6	0.006	$BaAl_4Sb_2O_{12}$	2.61	0.025
$Ca_3MgTiGe_3O_{12}$	2.45	0.08	$Na_3Al_2[PO_4]_2F_3$	1.93	0.03	$LiSc_2SbO_6$	2.23	0.02
$Ca_3MgSn[GeO_4]_3$	2.46	0.05	Cs_2KScF_6	1.74	0.05	$Na_3Sc_2Li_3F_{12}$	2.14	0.07
$Gd_3In_2Ga_3O_{12}$	2.31	0.09						

Table S2. Feature Set with importance score

Feature	Score	Feature	Score	Feature	Score
avg_Mulliken EN	7.9	avg_First ionization energy (kJ/mol)	6.1	$1/R^2$	5.2
avg_Metallic valence	5.2	avg_Martynov-Batsanov EN	4.6	beta	4.5
SGR No.	3.6	avg_Number of outer shell electrons	3.6	X	2.5
max_metal_ligand_bond_length	2.3	std_Mendeleev number	2.3	volume_per_atom	2.2
max_First ionization energy (kJ/mol)	2.2	volume_per_fu	2.0	polyhedron volume	2.0
std_Number of outer shell electrons	1.8	diff_Heat of fusion (kJ/mol)	1.8	avg_Boiling point (K)	1.8
diff_Heat atomization (kJ/mol)	1.7	Condensation	1.4	avg_Heat of fusion (kJ/mol)	1.4
R	1.3	std_Allen EN	1.3	max_Heat of fusion (kJ/mol)	1.0
distortion_index	1.0	std_Specific heat (J/g•K)	0.9	min_Melting point (K)	0.9
diff_Group number	0.8	avg_Pauling EN	0.8	(r)ionic_radii_difference	0.8
std_Gordy EN	0.8	volume	0.8	b	0.7
csm	0.7	mean_metal_ligand_bond_length	0.7	cation_site_madelung_potential	0.7
std_Boiling point (K)	0.6	c/a	0.6	b/a	0.6
std_Period number	0.6	max_Specific heat (J/g•K)	0.6	std_Density (g/mL)	0.6
std_Number of d electrons	0.6	std_Ionic radius (Å)	0.6	avg_Heat of vaporization (kJ/mol)	0.5
std_Number of valence electrons	0.5	a	0.5	c	0.4
min_metal_ligand_bond_length	0.4	diff_Crystal radius (Å)	0.4	avg_Zunger radius (Å)	0.4
diff_Number of valence electrons	0.4	avg_Melting point (K)	0.4	avg_Family number	0.4
std_Zunger radius (Å)	0.4	std_Mulliken EN	0.4	std_Pauling EN	0.4

avg_Ionic radius (Å)	0.4	diff_First ionization energy (kJ/mol)	0.4	a/b	0.3
std_Crystal radius (Å)	0.3	diff_Pauling EN	0.3	std_Covalent radius (Å)	0.3
avg_Density (g/mL)	0.3	avg_Number of p electrons	0.3	std_Heat of vaporization (kJ/mol)	0.3
std_L quantum number	0.3	avg_Specific heat (J/g•K)	0.3	diff_Thermal conductivity (W/m•K)	0.3
std_Thermal conductivity (W/m•K)	0.3	std_Group number	0.3	std_Family number	0.2
avg_Gordy EN	0.2	diff_Covalent radius (Å)	0.2	avg_Period number	0.2
std_Cohesive energy (eV)	0.2	std_Heat atomization (kJ/mol)	0.2	diff_Metallic valence	0.2
diff_Melting point (K)	0.2	diff_Ionic radius (Å)	0.2	std_First ionization energy (kJ/mol)	0.2
min_L quantum number	0.2	std_Melting point (K)	0.2	polar axis	0.2
std_Heat of fusion (kJ/mol)	0.2	min_Cohesive energy (eV)	0.2	diff_Boiling point (K)	0.2
Added_fe	0.2	inversion center	0.1	diff_Family number	0.1
max_Heat of vaporization (kJ/mol)	0.1	p3in7gr3up	0.1	std_Atomic number	0.1
spacegroup	0.1	std_Atomic radius (Å)	0.1	avg_L quantum number	0.1
density	0.1	Avg. cation electronegativity_27	0.1	std_Polarizability	0.1
max_Atomic number	0.1	avg_Polarizability	0.1	diff_Specific heat (J/g•K)	0.1
diff_Atomic weight	0.1	diff_Martynov-Batsanov EN	0.1	diff_Zunger radius (Å)	0.1
avg_Crystal radius (Å)	0.1	max_Boiling point (K)	0.1	min_Pauling EN	0.1
avg_Group number	0.1	avg_Covalent radius (Å)	0.1	diff_Atomic number	0.1
std_Number of p electrons	0.1	avg_Atomic radius (Å)	0.0	avg_Mendeleev number	0.0
max_L quantum number	0.0	max_Covalent radius (Å)	0.0	avg_Atomic weight	0.0
diff_Cohesive energy (eV)	0.0	gamma	0.0	Crystal system	0.0
std_Metallic valence	0.0	avg_Atomic number	0.0	diff_Number of s electrons	0.0
min_Heat of vaporization (kJ/mol)	0.0	avg_Number of s electrons	0.0	min_Boiling point (K)	0.0
min_Allen EN	0.0	std_Martynov-Batsanov EN	0.0	max_Metallic valence	0.0
diff_Gordy EN	0.0	max_Atomic radius (Å)	0.0	min_Atomic radius (Å)	0.0
min_Gordy EN	0.0	max_Atomic weight	0.0	max_Cohesive energy (eV)	0.0
max_Thermal conductivity (W/m•K)	0.0	max_Martynov-Batsanov EN	0.0	min_Number of valence electrons	0.0
avg_Allen EN	0.0	std_Atomic weight	0.0	min_Covalent radius (Å)	0.0
avg_Heat atomization (kJ/mol)	0.0	diff_Mulliken EN	0.0	diff_Period number	0.0
min_Atomic weight	0.0	min_Specific heat (J/g•K)	0.0	min_First ionization energy (kJ/mol)	0.0
avg_Cohesive energy (eV)	0.0	avg_Number of d electrons	0.0	diff_L quantum number	0.0
min_Crystal radius (Å)	0.0	diff_Allen EN	0.0	diff_Atomic radius (Å)	0.0
min_Mulliken EN	0.0	min_Polarizability	0.0	diff_Mendeleev number	0.0
avg_Number of valence electrons	0.0	diff_Density (g/mL)	0.0	max_Zunger radius (Å)	0.0
diff_Polarizability	0.0	max_Polarizability	0.0	max_Heat atomization (kJ/mol)	0.0
diff_Heat of vaporization (kJ/mol)	0.0	max_Number of valence electrons	0.0	max_Density (g/mL)	0.0
diff_Number of outer shell electrons	0.0	min_Martynov-Batsanov EN	0.0	max_Melting point (K)	0.0
min_Mendeleev number	0.0	diff_Number of d electrons	0.0	diff_Number of p electrons	0.0
alpha	0.0	std_Number of s electrons	0.0	Avg. anion polarizability_28	0.0

max_Family number	0.0	min_Number of s electrons	0.0	min_Heat atomization (kJ/mol)	0.0
max_Group number	0.0	max_Mendeleev number	0.0	max_Ionic radius (Å)	0.0
max_Crystal radius (Å)	0.0	max_Pauling EN	0.0	max_Gordy EN	0.0
max_Mulliken EN	0.0	max_Allen EN	0.0	max_Number of s electrons	0.0
max_Number of p electrons	0.0	max_Number of d electrons	0.0	max_Number of outer shell electrons	0.0
max_Period number	0.0	min_Thermal conductivity (W/m•K)	0.0	min_Atomic number	0.0
min_Period number	0.0	min_Group number	0.0	min_Zunger radius (Å)	0.0
min_Ionic radius (Å)	0.0	min_Metallic valence	0.0	min_Number of p electrons	0.0
min_Number of d electrons	0.0	min_Number of outer shell electrons	0.0	min_Density (g/mL)	0.0
min_Heat of fusion (kJ/mol)	0.0	min_Family number	0.0		

Table S3. Le Bail refinement statistics and refined unit cell parameters of host with 1% Cr^{3+}.

**Formula**	**Space Group:Z**	**a (Å)**	**b (Å)**	**c (Å)**	**Unit cell volume**	**wR (%)**
$Y_2Mg_3Ge_3O_{12}$	Fm-3m	12.2489(4)	12.2489(4)	12.2489(4)	1837.771(5)	7.0
$YInGe_2O_7$	C2/m	6.8427(5)	8.89430(15)	4.91051(23)	292.620(5)	9.46
$LiIIn_0W_2O_8$	2/m	9.5641(4)	11.5727(5)	4.95109(25)	547.903(7)	9.76
Gd_3SbO_7	Cmcm	7.5146(3)	10.6289(5)	7.5889(9)	606.15(8)	8.48
Ba_2ScTaO_6	Fm-3m	8.22198(20)	8.22198(20)	8.22198(20)	555.81(4)	9.28
Ba_2MgWO_6	Fm-3m	8.0999(4)	8.0999(4)	8.0999(4)	531.42(8)	21.7
$LiLaMgWO_6$	C2/m	7.8108(4)	7.82161(5)	7.8735(24)	481.015(18)	9.67
$Ca_3MgSi_2O_8$	C2/c	9.3351(13)	5.3066(7)	13.2904(20)	657.96(23)	9.75
